# Career intentions of final year medical students in Uganda after graduating: the burden of brain drain

**DOI:** 10.1186/s12909-015-0396-0

**Published:** 2015-08-01

**Authors:** Samuel Kizito, David Mukunya, Joyce Nakitende, Stella Nambasa, Adrian Nampogo, Robert Kalyesubula, Achilles Katamba, Nelson Sewankambo

**Affiliations:** 1 Clinical Epidemiology Unit, School of Medicine, College of Health Sciences, Makerere University, Kampala, Uganda; 2 Mbarara University of Science and Technology, Mbarara, Uganda

**Keywords:** Brain-drain, Doctors, Uganda

## Abstract

**Background:**

Uganda has severe shortage of human resources for health despite the heavy disease burden. The country has one of the highest fertility, and population growth rates in the world and is in dire need of trained health workers. The current doctor: patient ratio of 1:15000 is inadequate and this is further constrained by trained health workers leaving the country while others abandon the health sector. The aim of the study was to determine the career intentions of the final year medical students to leave the county and health field after graduating and the associated factors.

**Methods:**

We conducted a cross-sectional study among 251 final year medical students from Makerere, Mbarara, Gulu and Kampala International Universities. We enrolled all the eligible final year medical students. The study was conducted using face-to-face questionnaires in each university. We determined the demographics, reasons for leaving the country and health sector and the intended destinations of medical students who planned to leave the country. Data was entered in Epidata then exported and analyzed in stata 12.

**Results:**

Of the 251 students enrolled in the study, 28(11.2 %) wanted to leave the health sector, with Mbarara University of Science and Technology (MUST) having the highest percentage, 16.7 % and Kampala International University (KIU) the least, 7.7 %. Of the 28 who intended to leave the health sector, 82.1 % wanted to join the business sector, 10.7 % agriculture, and 7.1 % politics. Reasons given for the intent to leave were; lack of equipment and supplies in hospitals, over whelming patient numbers, very risky working environment, low payment to doctors, and political reasons. Overall, 112 (44.6 %) of the participants wanted to leave the country with 30.3 % intending to migrate to United States of America (USA), 11.9 % to United Kingdom (UK), 11.0 % to South Africa among others. Some of the reasons given were; doctors are paid a high salary abroad, safe working environment, and desire to continue academics. Age was associated with intention to leave the country (OR = 1.64; 95 % CI: 1.00 – 4.82).

**Conclusions:**

In a country in dire need of health workers, the study showed high proportion of trainee health workers planning to abandon their professions or emigrate from Uganda after training.

## Background

There is a critical shortage of health workforce in Africa especially in the sub-Saharan region [1–4], estimated at 817,992 [3, 5]. While Africa accounts for more than 24 % of the global disease burden, it has only 3 % of the world’s health workers and spends less than one percent of total global resources dedicated to health. This shortage is worsened by the emigration of doctors [6]. The magnitude of brain drain in Africa has been increasing alarmingly in the past decades [6, 7]. Emigration is likely to lead to deterioration in the working conditions of the doctors who stay behind as it increases the workload for the remaining health workers, compromising access to and quality of health care to the population, and impairs the ability of the health care system to achieve the desired population health objectives [8]. For efficient and effective operation, the health sector requires skilled and competent personnel and as such the emigration of doctors affects the overall effectiveness of the health sector [9, 10]. The doctor: patient ratio in Uganda is 1:15000 [11, 12], which implies that most of the general population may not easily access the much needed basic health care.

Between 2005 and 2011, at least 210 doctors emigrated from Uganda of whom 67.6 % left for work elsewhere, and the others for further studies. Of the 210 doctors who emigrated, 29.0 % left for another African country and the rest for overseas destinations [13]. Several reasons have been advanced as the leading causes for the emigration of doctors. These include “push” and “pull” factors [14]. Identified “push” factors included lack of or poor post-graduate training opportunities, poor working conditions, civil unrest and personal security [15–18]. The “pull” factors include international opportunities for career advancement, greater financial rewards and better working conditions [8, 17, 18]. The sending country incurs two forms of losses; the resources spent in educating the doctor, and the lost value of health care services the doctor would have rendered [2, 8, 19]. It is estimated that the total cost of educating a doctor in an African setting from primary school to graduation from university is about US $66,000 [19, 20] and for every doctor that emigrates, the country loses an equivalent of approximately US $1,900,000 [21].

Uganda has a large and growing population, a huge disease burden and very limited resources including well-trained health workers. In 2006, the Global Health workforce alliance was formed to identify, implement and advocate solutions to the crisis. In 2010, the World Health Organization (WHO) generated a global Code of Practice with the aim of finding solutions to international migration of health professionals. All these strategies were generated without putting into consideration the students’ career inclinations. The migration is still occurring [4]. There is thus need to understand the extent of and reasons for intentions to migrate, and the likely destinations of the future doctors in Uganda if the above strategies are to be successfully implemented. Previous studies have mainly centered on high income countries, and those carried out in low resource environments have focused on graduates and practicing doctors. There is no reliable information with regards to students’ career intentions in low resource environments. Thus our major aim was to explore: 1.) How many students intended to leave the health sector, 2.) How many students intended to migrate after graduation, 3.) what were the destinations for the students that leave the country and 4.) What factors were associated with students’ leaving the country and health sector after graduating.

## Methods

### Study design and setting

This was a cross-sectional study of Ugandan undergraduate medical students in their fifth and final year of studies at the four medical universities in Uganda; Makerere University College of Health Sciences (MakCHS), Mbarara University (MUST), Gulu University and Kampala International University (KIU). The study enrolled 251 participants and all of these completely answered the semi-structured questionnaires that were used to collect the data. The tool was pretested on 11 non-final year medical students. They were distributed to the students who accepted to participate in the study and provided written informed consent. They were requested to complete them at their convenience. In each of the universities, there was a focal point person who coordinated the process of data collection.

### Study variables

Data collected included background information like sex, age, marital status, family income, and academic performance assessed using the Cumulative Grade Progress Average (CGPA).

The questionnaire also ascertained for students intention to work in health sector or otherwise. It was adapted from a previous study among nursing students in Uganda [22]. Students were asked: what factors could influence you to leave the health sector? And the possible responses included: poor working conditions, inadequate supply of equipment, patient overload, low salaries, lack of mentorship, the respect that doctors command and others, specify.

To determine the factors likely to influence the students’ intention to work or leave the country, we asked the students: what factors could encourage you to work abroad? The answering responses were; salary, social/family support, good working conditions, political stability, and better academic opportunities abroad. Special attention was given to factors that encourage students to stay and practice from Uganda; the students who intended to stay were asked: what factors could encourage them to stay and work in Uganda: the answering possibilities were; racism abroad, high cost of living, long emigration process and lack of family and social support abroad, specify others.

### Ethics of the study

Approval to conduct the study was obtained from the Makerere University College of Health Sciences, School of Medicine Research and Ethics Committee. Written informed consent was provided by each of the participants before they were enrolled into the study.

### Data management and analysis

The data were entered in Epidata Version 3.1, checked, and frozen. It was then exported to stata version 12 for analysis. Data was adjusted for clustering at the level of university. Demographic data was presented as means and percentages. Comparisons were made between students who intended to leave the country and those who intended to stay. For the categorical variables, comparisons were made using chi-squares or Fisher’s exact test while for the continuous data, student t test was used for the parametric data and the Mann Whitney U test for the non-parametric data. The outcome was dichotomized into staying or leaving the country, and staying or leaving the health sectors. Logistic regression models were then run to assess factors associated with leaving the country, and the health sector respectively. Factors that were entered in the multivariate model were based on p value less than 0.2 or less at bivariate analysis previous literature as well as factors shown to significantly influence migration and career choice according to previous literature. Confounding factors were assessed at a 10 % difference between the odds ratios for the unadjusted and adjusted models. The goodness of fit of the final model was assessed using the Hosmer and Lemeshow statistic.

## Results

### Demographics

Of the 251 students Makerere university provided the largest number (99/251 (39.4 %)) while KIU provided the least, 39 (15.5 %). As shown in Table 1, most of the students (91.2 %) were single, mean age was 25.9 years (SD 0.6). Overall, 87.9 % of the students perceived their socio-economic status as being in the middle class.

**Table 1:** Demographic characteristics of 251 final year medical students in Uganda’s universities, 2012–13

Variable	Categories	N (251)	Percentage
Institution:	MakCHS^*^	99	39.4 %
	MUST^**^	54	21.5 %
	Gulu	59	23.5 %
	KIU^***^	39	15.5 %
Sex	females	66	26.3 %
	males	185	73.7 %
Marital status	Single	229	91.2 %
	Married	20	8.0 %
	Divorced	1	0.4 %
	Widowed	1	0.4 %
Perceived Socio-Economic Status	Poor	22	8.8 %
	Low-middle class	135	54.2 %
	Upper middle class	84	33.7 %
	Upper class	8	3.2 %

^*^Makerere University College of Health sciences

^**^Mbarara University of Science and Technology

^***^Kampala International University

Close to three quarters of the study participants were males (185/251 (73.7 %)). MakCHS had 31.3 % females, MUST 29.6 % females, Gulu 27.1 % females though KIU had only 7.7 % females.

### Intention to leave the health sector

Overall 28/251, 11.2 % (7.13 – 16.3) of the students had the intention of leaving the health sector, with MUST having the highest percentage (16.7 %), and KIU having the least (7.7 %) while 9 (16.7 %) and 8 (13.6 %) were from MUST and Gulu respectively. Details are highlighted in Table 2.

**Table 2:** Final year medical students intending to leave Uganda after graduating, 2012–13 (*n* = 251)

Variable	Categories	Number intending to leave (*n* = 112)	Percentage
University	MakCHS^*^	40/99	40.4
	MUST^**^	26/54	48.1
	Gulu	25/59	42.4
	KIU^***^	21/39	53.8
Reason for leaving^****^	High salary abroad	84/112	75.0
	Good work conditions	60/112	53.6
	Political stability	41/112	36.6
	Go for further studies	65/112	58.0
Factors for staying^****^	Factors that limit going		
	Racism abroad	38/139	27.3
	High cost of limiting abroad	46/139	33.1
	Long emigration process	50/139	36.0
	Lack of family support	41/139	29.5
	Factors that encourage staying		
	Reward for government scholarship	58/139	41.7
	Satisfied with working conditions	17/139	12.2

^*^Makerere University College of Health sciences

^**^Mbarara University of Science and Technology

^***^Kampala International University

^****^some students gave more than one reason

Of those students that intended to leave the health sector, 23/28 (82.1 %) wanted to join business, 3 (10.7 %) agriculture, and 2(7.1 %) politics.

Reasons given by the students for intention to leave the health sector included; lack of equipment and supplies in hospitals, overwhelming patient numbers, very risky working environment, low payment to doctors, and political reasons.

As highlighted in Table 3, at multivariate analysis, factors that were associated with leaving the health included: CGPA (OR = 0.30; 95 % CI: 0.10 – 0.88), having the intention to leave the country (OR = 3.22; 95 % CI: 1.15 – 9.06) being of lower middle and upper-middle socio-economic status respectively compared to lower class (OR = 0.15; 95 % CI: 0.04 – 0.67 and OR = 0.21; 95 % CI: 0.05 – 0.94) respectively.

**Table 3:** Factors associated with medical students’ intention to leave the health sector in Uganda after graduation, 2013. (*n* = 255)

		Bivariate analysis		Multivariate analysis	
Variable	Categories	OR (95 % CI)	P value	OR (95 % CI)	P value
Institution	MakCHS^*^	1		1	
	MUST^**^	1.82 (0.69 – 4.80)	0.226	1.09 (0.37 – 3.20)	0.874
	Gulu	1.61 (0.61 – 4.21)	0.335		
	KIU^***^	0.76 (0.20 – 2.92)	0.987	0.41 (0.03 – 6.06)	0.520
Age		0.96 (0.86 – 1.08)	0.516		
Sex	Female	1		1	
	Male	1.01 (0.43 – 2.38)	0.981	0.89 (0.30 – 2.66)	0.833
Marital status	Single	1		1	
	Married	0.60 (0.19 – 1.92)	0.393	0.50 (0.11 – 2.33)	0.379
Perceived social status	Lower class	1		1	
	Lower middle	0.33 (0.10 – 1.06)	0.062	0.15 (0.04 – 0.67)	0.012
	Upper middle	0.36 (0.11 – 1.25)	0.108	0.21 (0.05 – 0.94)	0.041
	Upper class	3.40 (0.62 – 18.75)	0.160	2.38 (0.36 – 15.6)	0.365
CGPA^****^		0.78 (0.35– 1.74)	0.543	0.30 (0.10 – 0.88)	0.029
Want to leave country		2.95 (1.28 – 6.81)	0.011	3.22 (1.15 – 9.06)	0.026

^*^Makerere University College of Health sciences

^**^Mbarara University of Science and Technology

^***^Kampala International University

^****^Cumulative Grade Progress Average

### Intention to leave the country after graduation

Overall, 112/251, 44.6 % (38.7 – 61.3) of the students had the intention of leaving the country after graduation. Among females 38/66 (57.6 %) intended to stay in Uganda after graduating compared to 101/185(54.6 %) of the males. KIU which has a high proportion of foreign students had the highest percentage of students intending to leave the country, 21/39(53.8 %) while MakCHS had the least, 40/99 (40.4 %), as shown in Table 3. Major factors that stood out as reasons for the intention to emigrate after graduation included; doctors being paid a higher salary abroad, 84/112(75.0 %), safe/good working environment, 60/112(53.6 %), and desire to continue with academics 65/112 (58.0 %). Of the students who had the intention to leave the country, close to one third (36/118 (30.3 %)) mentioned USA as their intended destination, 14 (11.9 %) UK, 13 (11.0 %) South Africa, followed by Canada and Rwanda with 7(5.9 %) each and 22.0 % did not specifying their intended country of destination (Table. 2). Other countries that were mentioned as destination countries were: Australia, France, Germany, Kenya, Sudan, Botswana, Brazil and Israel. Some students had more than one country as the intended destination.

The reasons given by the students who opted to stay in the country included; family and social ties or influence in Uganda, paying back to the government for sponsoring the student’s education, lack of easy means of living the country, and 17 students were opting to stay and work in Uganda because they were satisfied with the working conditions in Uganda.

A comparison showed that those who intended to stay in the country include significantly more people who want to stay in health field work (130/143, 93.5 %) than do those that intended to leave (93/112, 82.3 %) , chi-square = 6.886, p value 0.017. there were no significant differences between these groups in other characteristic variables mentioned in Table 3.

At multivariate analysis, the only factor significantly associated with leaving the country was age (OR = 1.64; 95 % CI: 1.00 – 4.82). Details are shown in Table 4.

**Table 4:** Factors associated with medical students’ intention to leave Uganda after graduation, 2013. ( *n* = 255)

		Bivariate analysis		Multivariate analysis	
Variable	Categories	OR (95 % CI)	P value	OR (95 % CI)	P value
Age		1.01 (0.98 – 1.03)	0.623	1.64 (1.00 – 4.82)	0.050
Sex	Female	1		1	
	Male	1.14 (0.65 – 2.01)	0.646	0.05 (0.001 -1.59)	0.087
CGPA^****^		0.98 (0.89 – 1.07)	0.626		
Perceived social status	Lower class	1		1	
	Lower middle	0.40 (0.16 – 1.01)	0.053	0.02 (0.001 -15.0)	0.235
	Upper middle	0.45 (0.17 – 1.18)	0.105	0.03 (0.001 – 4.88)	0.175
	Upper class	0.95 (0.73 – 4.17)	0.957		
Not satisfied with work conditions		1.20 (0.55 – 2.62)	0.640	33.6 (0.5 – 2313.6)	0.104
Said hospital supplies inadequate		1.13 (0.59 – 2.17)	0.704	0.92 (0.04 – 19.91)	0.958
Said patient load is high		1.07 (0.60 – 1.89)	0.830	0.1 (0.01 – 2.57)	0.108
Said payment is low		1.44 (0.37 – 5.53)	0.591		
Institution affiliation	MakCHS^*^	1		1	
	MUST^**^	1.34 (0.69 – 2.60)	0.394	1.73 (0.07 – 44.73)	0.740
	Gulu	1.06 (0.55 – 2.03)	0.865		
	KIU^***^	1.68 (0.80 – 3.54)	0.173	0.04 (0.003-4.90)	0.190
Intends to leave health sector		2.95 (1.28 – 6.81)	0.011		

^*^Makerere University College of Health sciences

^**^Mbarara University of Science and Technology

^***^Kampala International University

^****^Cumulative Grade Progress Average

## Discussion

It is gratifying that there are still some students who intend to stay and work in the country. However it is a source of concern that close to one half of the final year medical students trained in Uganda have intentions of leaving the country after graduating. These figures are higher than those reported in other developing countries [23, 24]. The relatively high percentages in KIU could probably be due to the influence from the high number of international students at the university towards the local students. Makerere University has a different curriculum compared to the other medical schools and its students participate in community based education for more than a month during each of the 5 years of medical training. This might contribute to enhancing students’ appreciation of the health needs of the community, and probably instills a spirit of wanting to stay and serve the country [25]. In the rest of the medical universities, the Community Based Education and Services (COBES) program runs for about a month during the entire 5 years of training. Studies have suggested earlier that students who have received substantial training based in rural areas have high chances of being retained and work in the country and that this likelihood correlates with the time of training spent in rural areas [4, 25].

Close to one third of all the students intending to leave the country, had USA as the intended destination, followed by UK. Both are stable developed countries, have advanced medical technology, and much greater availability of resources than Uganda. Only very few students had another African country or a developing country as their destination. This may be due to the fact that these countries share similar problems with Uganda and therefore students consider them less attractive destinations.

The reasons for the students’ decision to leave the health sector or the country were not much different from those found in previous studies [15, 26–28]. According to a WHO report the shortage of the global health workforce was attributed to insufficient recruitment and retention, poor working conditions and inadequate remuneration [29]. By 2009, although the cost of living in Uganda had more than tripled, the salary scales had remained stagnant for more than two decades [30]. Compared to the neighboring East African countries, Ugandan doctors receive the lowest salary [31, 32].

Only 12.23 % were intending to stay because they were satisfied with working condition. These numbers are smaller than those demonstrated in previous studies [33]. Students who wanted to leave the country were more than three times likely to leave the health sector and students of lower socio economic status were more likely to leave the health sector. Other major factors for leaving were the good and safe working conditions abroad and this is also consistent with the earlier studies [8, 10, 17, 18, 27–29]. More than ten percent of the students intended to leave the health sector, and not surprisingly the majority of them intended to join business. This internal brain drain has been demonstrated in other studies [1, 32]. This may in part indicate that the payments that the doctors are receiving are not satisfactory [32] and not motivating the future generation. Whereas medical universities have a role to play in influencing students’ attitudes about working in their home country there are many other extraneous factors that influence the trainees’ decisions [2].

## Conclusions

The study has shown the high intention of trainee health workers to abandon the profession and to leave the country after training. Not so many of their final year medical students intend to work in the Ugandan health care. Salaries seem to be an important factor in influencing the decision to leave. As a result of the major limitations in this study, bigger studies are recommended.

### Limitations

The study had a small number of participants therefore the possibility of a type 2 error cannot be ruled out. Additionally the study looks at the intentions, which may not necessarily translate into actions.

## Abbreviations

COBES: Community based education and services
FGD: Focus group discussion
HIV: Human immunodeficiency virus
KIU: Kampala International University
MakCHS: Makerere University College of Health Sciences
MDGs: Millennium development goals
MUST: Mbarara University of Science and Technology
TB: Tuberculosis
UK: United Kingdom
USA: United States of America
